# Coding region structural heterogeneity and turnover of transcription start sites contribute to divergence in expression between duplicate genes

**DOI:** 10.1186/gb-2009-10-1-r10

**Published:** 2009-01-28

**Authors:** Chungoo Park, Kateryna D Makova

**Affiliations:** 1 Center for Comparative Genomics and Bioinformatics, Department of Biology, The Pennsylvania State University, University Park, PA 16802, USA

## Abstract

**Background:**

Gene expression divergence is one manifestation of functional differences between duplicate genes. Although rapid accumulation of expression divergence between duplicate gene copies has been observed, the driving mechanisms behind this phenomenon have not been explored in detail.

**Results:**

We examine which factors influence expression divergence between human duplicate genes, utilizing the latest genome-wide data sets. We conclude that the turnover of transcription start sites between duplicate genes occurs rapidly after gene duplication and that gene pairs with shared transcription start sites have significantly higher expression similarity than those without shared transcription start sites. Moreover, we find that most (55%) duplicate gene pairs do not retain the same coding sequence structure between the two duplicate copies and this also contributes to divergence in their expression. Furthermore, the proportion of aligned sequences in *cis*-regulatory regions between the two copies is positively correlated with expression similarity. Surprisingly, we find no effect of copy-specific transposable element insertions on the divergence of duplicate gene expression.

**Conclusions:**

Our results suggest that turnover of transcription start sites, structural heterogeneity of coding sequences, and divergence of *cis*-regulatory regions between copies play a pivotal role in determining the expression divergence of duplicate genes.

## Abstract 2

Gene expression data for duplicated gene pairs in humans provides insights into the regulatory factors affecting the expression divergence of these genes and implications for their evolution.

## Background

Because of the importance of gene duplication in evolution [1-5], it is crucial to know how duplicate genes diverge and which factors determine their destiny. Recently, genome-wide analyses of microarray data [6] have revealed patterns of expression divergence in duplicate genes, which are necessary for understanding the emergence of new functions after gene duplication. Numerous studies indicated that genes diverge rapidly in their expression after duplication [7-12]. Population genetic models proposed directional selection and relaxation of selective constraints as possible forces driving the evolution of expression in duplicate genes, although the relative frequency of these two scenarios in the evolution of paralogs is still being debated [4,5,13]. These population genetic models have been implemented under the assumption that two duplicated gene copies are structurally and functionally identical immediately after duplication. However, this assumption is sometimes violated. First, genes duplicated via retrotransposition lose regulatory sequences and include additional sequences at each side (for example, poly(A) tails at 3' terminus and short direct repeats at both termini), so that retrotransposed copies differ from the corresponding parental genes [4,13,14]. Second, tandem duplication by unequal crossing over might not include the entire coding sequence and/or regulatory elements specifying expression of a parental gene. Indeed, Katju and Lynch [15] demonstrated that more than half of newborn duplicate genes in *Caenorhabditis elegans *represent not complete, but rather partial or chimeric duplications. Such structural heterogeneity may play an important role in rapid expression divergence between human duplicate genes as well; however, it has not been considered in detail in previous studies.

Transposable elements (TEs) represent another factor that might account for the expression divergence of duplicate genes, since several studies provided evidence of TEs altering gene expression. Jordan and colleagues [16] showed that almost 25% of human promoter regions as well as many other *cis*-regulatory elements contain, or at least overlap with, TE-derived sequences. This result was later confirmed by another study [17]. A specific example of the importance of TEs in the regulation of gene expression comes from the *CYP19 *gene, which encodes the aromatase enzyme, important for estrogen biosynthesis [18]. Because of the recent insertion of a long terminal repeat into the first exon of one of the isoforms of human *CYP19*, the gene gained expression in placenta, while its mouse ortholog has no long terminal repeat and is not expressed there [19].

Finally, alternative promoter usage by duplicate genes should be considered as a mechanism for rapid expression divergence. Recent comprehensive studies concluded that many known genes in the human genome are expressed from alternative promoters [20-23]. Similarly, approximately 22% of genes in the ENCODE regions have functional alternative promoters [24]. The alternative promoters provide a heterogeneity in tissue-specific expression patterns and levels, developmental activity, and translational efficiency [25-27]. As a result, the use of alternative promoters might be one of the major sources for achieving transcriptome diversity and one of the routes by which duplicate genes acquire divergence in their expression.

To investigate what drives expression divergence of human paralogs on a genome-wide scale, we addressed the following three questions in the present study: how frequently the turnover of transcription start sites (TSSs) occurs between duplicate genes; how often duplicate gene copies (their coding sequences) differ from each other structurally; and whether the density of copy-specific TEs within *cis*-regulatory regions influences expression divergence in duplicated genes. We utilized the gene expression profile available for 61 non-redundant and non-pathogenic human tissues [28], the largest comprehensive expression profile of human genes available to date, and assessed the contributions of TSS turnover, coding sequence structural heterogeneity, and TE integration to divergence in duplicate gene expression.

## Results

### Identification of duplicate genes

Utilizing two different methods, FASTA and TRIBE-MCL, we identified 6,536 and 7,027 non-redundant human duplicate gene pairs, respectively (see Materials and methods for details). These pairs represented 3,313 and 3,555 gene families, respectively. After filtering out duplicate gene pairs with synonymous rate (*K*_*S*_) >2 and/or lacking a start codon, we obtained 2,790 and 2,750 duplicate gene pairs using the former and the latter methods, respectively. A total of 1,600 duplicate gene pairs overlapped between these two data sets (Additional data file 2). All subsequent analyses were carried out for duplicate genes identified with each of the two methods. Because the results were similar, we present the results only for duplicate genes identified with the FASTA method (2,790 gene pairs in group A), as this method is stricter for clustering proteins into families compared with the TRIBE-MCL method [29,30].

From human U133A and GNF1H oligonucleotide arrays [28], we defined 14,505 genes that mapped to probes with a one-to-one correspondence (see Materials and methods), thus minimizing cross-hybridization. Among these genes, we were able to detect 2,924 non-redundant duplicate gene pairs belonging to 1,792 multiple gene families. After filtering out duplicate gene pairs with *K*_*S *_>2 and/or lacking a start codon, we obtained 1,015 duplicate gene pairs (group B, representing a subset of group A). In the remainder of the manuscript, we consider duplicate genes of group B when gene expression is investigated and duplicate genes of group A otherwise.

### Turnover of TSSs between duplicate genes

Initially, we analyzed the divergence in the position of TSSs between copies in each duplicate gene pair. Using tag clusters, which were built by grouping overlapping tags (namely, 5'-end-sequences) with the same strand, from large-scale tag clustering of the cap analysis of gene expression (CAGE) [20] and the paired-end ditags (PETs) [31], putative TSSs of each gene were identified (see Materials and methods). From 2,790 duplicate gene pairs in group A, we excluded duplicate gene pairs that were duplicated by retrotransposition or for which at least one copy lacked a TSS(s) identified by either CAGE or PETs. As a result, 1,124 duplicate gene pairs were retained. To evaluate sharing of TSSs between duplicate genes, we compared the sequences of genomic regions surrounding putative TSSs (as identified by CAGE or PETs) between the two copies for each of these 1,124 duplicate gene pairs. We considered 110 bp (-20 bp to +90 bp) surrounding each TSS (later called the 'TSS region'), because there was a clear peak in the average sequence similarity between TSSs of duplicate genes in this region (Additional data file 3) and because several studies indicated that a region of this size surrounding TSSs was well conserved between human and mouse orthologs [32,33]. Sequence similarity between all possible combinations of TSS regions from each duplicate gene pair was considered. If at least one pair of TSS regions had an identity greater than 60%, it was defined as a TSS(s) shared between the two duplicate copies. As a result, 13.6% (153 out of 1,124) of duplicate gene pairs had shared TSSs.

**Figure 1:**
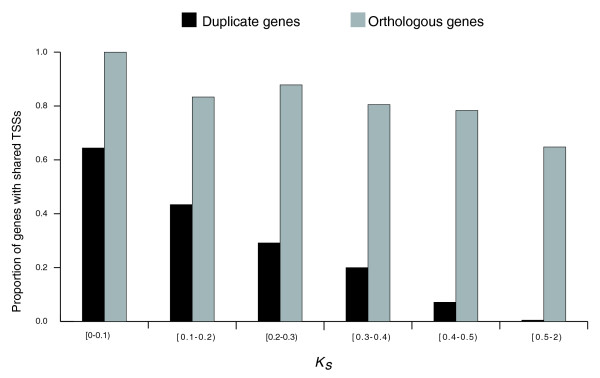
The decline in the proportion of group A duplicate gene pairs with shared TSSs (shown in black) depending on the time since duplication (approximated by *K*_*S*_). The proportion of human-mouse orthologous genes with conserved TSSs is shown for comparison (in gray); in this case variation in *K*_*S *_is due to regional variation in substitution rates.

We observed that the relative frequency of gene pairs with shared TSSs decreases with increasing *K*_*S*_, a proxy of time since duplication (Figure 1). The L-shaped distribution observed in Figure 1 implies a rapid turnover of TSSs after gene duplication. Already at *K*_*S *_= 0.1, corresponding to only about 33 million years ago since duplication [34], a mere 64% of duplicate genes share TSSs. Considering an instantaneous *K*_*S *_rate according to [35] did not alter our results (Additional data file 4).

Interestingly, the turnover of TSSs between human duplicate genes was much more rapid than between human-mouse orthologs. Indeed, for 1,610 human-mouse orthologs considered (see Materials and methods), the mean *K*_*S *_was 0.61 (with a 95% confidence interval of 0.60-0.63), while the proportion of orthologs with shared TSSs was 0.71, several fold higher than the proportion of human duplicate genes with similar *K*_*S *_(Figure 1).

To estimate the relationship between TSS usage patterns (for example, shared TSSs versus non-shared TSSs) and gene duplication mechanisms, the duplicate genes were divided into three classes: retrotransposed duplicate genes, tandem, and nontandem duplications (see Materials and methods for details). The relative frequencies of gene pairs with shared TSSs in each class were calculated (thus, we analyzed 1,124 non-retransposed genes as above plus 220 retrotransposed genes). Duplicate gene copies in which one of the pair has one exon and the duplicate copy has multiple exons were called retrotransposed duplicate gene copies. We found that among paralogs with shared TSSs, the majority of pairs represented tandem duplicates (Additional data file 1).

Interestingly, about 30% (67 out of 220) of retrotransposed duplicate gene pairs retained the same TSSs (Additional data file 1). To evaluate whether the retrotransposed gene pairs with shared TSSs tend to undergo stronger purifying selection than those without shared TSSs, the median nonsynonymous-to-synonymous rate ratios (*K*_*A*_/*K*_*S*_) were compared between these two groups of genes; however, no significant difference was detected (0.475 versus 0.499; *P *> 0.1, Mann-Whitney U test).

Next, to test whether the turnover of TSSs may contribute to the expression divergence in duplicate genes, the Pearson correlation coefficient of expression values (*R*_*expression*_; calculated for 61 non-redundant tissues) between the two copies in each pair was computed and compared among group B duplicate gene pairs with shared TSSs versus those without shared TSSs (a total of 581 group B pairs with available TSS data were included in the analysis). Duplicate genes with shared TSSs had significantly higher *R*_*expression *_values than those without shared TSSs (0.437 versus 0.080; *P *< 0.01, Mann-Whitney U test). It is conceivable that the significant difference in *R*_*expression *_values is due to different synonymous rates in genes with shared TSSs versus those without shared TSSs. Indeed, we observed that all duplicate genes (belonging to group B) with shared TSSs had *K*_*S *_<0.4, while more than 97% of gene pairs without shared TSSs had *K*_*S *_≥ 0.4. However, if only genes with *K*_*S *_<0.4 were considered, the gene pairs with shared TSSs still had higher (but not significantly so) *R*_*expression *_values than those without shared TSSs (0.437 versus 0.140; *P *> 0.05, Mann-Whitney U test).

The 60% identity threshold among the TSS regions that was tentatively inferred from substitution rates between human and mouse ortholog core promoters [36] may be inadequate for estimating the sharing of TSSs among human paralogous genes. Thus, we reclassified the sharing of TSSs between copies of duplicate genes using several identity thresholds (40%, 50%, 70%, and 80%). Although the numbers of duplicate genes with shared TSSs in each bin varied with the threshold, the frequency of gene pairs with shared TSSs decreased over divergent time independent of the threshold used (Additional data file 5), consistent with the pattern observed with the 60% identity threshold (Figure 1). Moreover, regardless of the identity threshold, the *R*_*expression *_values were significantly higher in duplicate genes with shared TSSs versus those without shared TSSs (data not shown).

### Structural heterogeneity in coding regions of human duplicate genes

By reconstructing the full-length coding sequences via concatenating exons from multiple splicing variants for each gene separately, each pair of duplicate genes was classified into one of two structural categories: completely similar and incompletely similar. If the proportion of aligned sequences was greater than 0.9, duplicate gene pairs were categorized as completely similar and as incompletely similar otherwise. For some analyses, incompletely similar duplicate gene copies were classified in one of the three non-overlapping groups: 5' similar, 3' similar, and neither 5' nor 3' similar. If alignments between the two copies started at the start codons of both copies, then such duplicates were classified as 5' similar. Alternatively, if the alignments ended at the stop codons of both copies, we classified the duplicate genes as 3' similar. The remaining duplicate gene pairs were labeled as neither 5' nor 3' similar.

**Figure 2:**
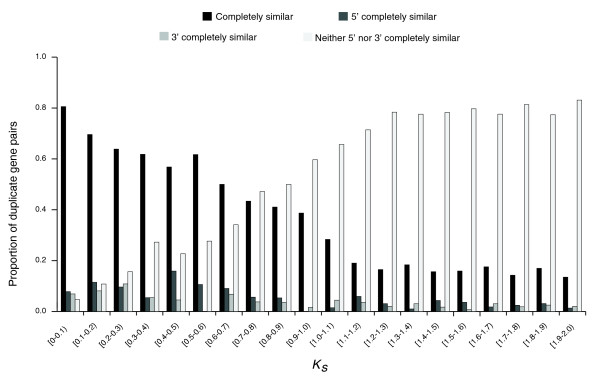
Proportion of group A duplicate gene pairs classified by coding sequence structural heterogeneity.

After excluding genes that lacked start/stop codons or consensus splice sites, 2,591 duplicate gene pairs were retained (from 2,790 pairs of group A; for group B, 889 duplicate gene pairs were retained). We found that 55% (1,429 out of 2,591) of duplicate gene pairs had incompletely similar structures. As expected from the divergence of the coding sequence over time, the proportion of duplicate gene pairs with completely similar structures decreased gradually with divergence between the two duplicate copies, approximated by *K*_*S *_(Figure 2). Considering an instantaneous *K*_*S *_rate according to [35] did not alter our results (Additional data file 6). Interestingly, even at the smallest duplicate gene divergence (*K*_*S *_<0.1), the proportion of genes with completely similar structures was only 80% (Figure 2). Although this finding might be affected by misannotations, our results suggest that some duplicate genes might have acquired structural differences during duplication.

**Figure 3:**
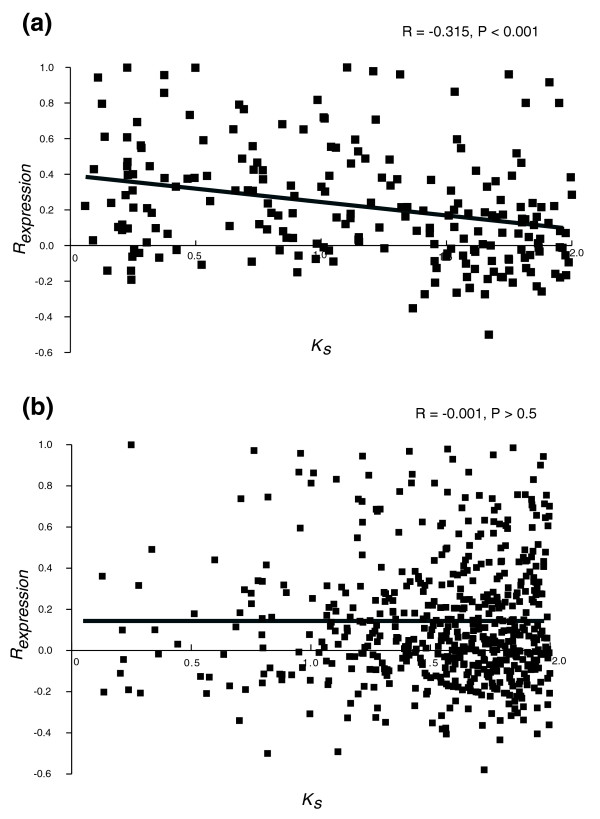
The relationship between *K*_*S *_and *R*_*expression *_for group B duplicate genes with **(a)** completely similar structures and **(b)** incompletely similar structures.

To analyze whether the incompletely similar structures of duplicate genes can lead to expression divergence, we compared the relationship between *R*_*expression *_and *K*_*S *_for duplicate genes with completely versus incompletely similar structures. Before addressing this issue, retrotransposed duplicate genes (a total of 108 out of 889 genes retained in group B) were excluded because, as retrotransposition does not include a promoter, it can lead to expression divergence regardless of structural heterogeneity in coding sequence between duplicates. We found that: the correlation coefficient between *R*_*expression *_and *K*_*S *_for duplicate gene pairs with completely similar structures was significantly lower than that for pairs with incompletely similar structures (R = -0.315 versus R = -0.001; Fisher's z test, z = -4.028, *P *< 0.001; Kolmogorov-Smirnov test for normality, *P *< 0.010; Figure 3 and Table 1); and duplicate genes with completely similar structures had significantly higher y-intercepts of regression lines than duplicate genes with incompletely similar structures (0.407 versus 0.134; z = 2.672, *P *< 0.01). These observations suggest that, immediately after duplication, the expression pattern is more similar for duplicate gene pairs retaining the same versus acquiring different coding sequence structures, and that divergence of gene expression is more dependent on evolutionary time for duplicate gene pairs with completely versus incompletely similar structures. To estimate the importance of sharing of 5' regions of coding sequences between duplicate gene copies, which can be an indirect indicator of common transcription regulation mechanisms, we separately considered duplicate gene pairs completely similar at the 5' end only (a total of 24 gene pairs from group B that were otherwise genes with incompletely similar structures) and calculated the correlation coefficient between their *R*_*expression *_and *K*_*S*_. The correlation was negative, but not significant (Table 1). When duplicate gene pairs having completely similar and 5' similar structures were considered together, the correlation coefficient between *R*_*expression *_and *K*_*S *_was somewhat lower than that for duplicate gene pairs with completely similar structures (Table 1), although the difference was not significant (z = -0.093, *P *> 0.1). We observed that there was no correlation between *R*_*expression *_and *K*_*S *_for duplicate genes with 3' similar structure and with neither 5' nor 3' similar structure (Table 1). These results suggest that maintenance of the entire coding region (and not just of its 5' or 3' portion) is important for determining gene expression profile after duplication.

**Table 1:** The relationship between *K*_*S *_and *R*_*expression *_in each structural category using group B duplicate gene pairs

Structural categories	Number of gene pairs	*K*_*A*_/*K*_S_*	*K*_S_*	*R*_expression_*	Pearson correlation coefficient of *K*_*S *_versus *R*_*expression *_(*P*-value)
Completely similar	214	0.296 (0.237)	1.153 (1.225)	0.213 (0.162)	-0.315 (<0.001)
5' similar	24	0.391 (0.311)	1.292 (1.501)	0.053 (0.026)	-0.157 (NS)
3' similar	23	0.302 (0.311)	1.365 (1.610)	0.346 (0.249)	0.019 (NS)
Neither 5' nor 3' similar	520	0.551 (0.456)	1.565 (1.658)	0.126 (0.063)	0.017 (NS)
Incompletely similar (the sum of the above three categories)	567	0.534 (0.444)	1.545 (1.646)	0.132 (0.068)	-0.001 (NS)
Completely and 5' similar	238	0.307 (0.246)	1.167 (1.263)	0.197 (0.151)	-0.307 (<0.001)

*Values are mean (median). NS, not significant.

To estimate differences in selective pressure among duplicate genes in different structural categories, their *K*_*A*_/*K*_*S *_ratios were compared (Table 1). We observed that *K*_*A*_/*K*_*S *_was significantly lower for duplicate genes with completely similar structures than for those with incompletely similar structures (*P *< 0.001, Mann-Whitney U test; Table 1), suggesting that the former genes are subject to stronger purifying selection than the latter genes.

### Divergence of *cis*-regulatory sequences between duplicate genes

Next, we evaluated the relative contribution of *cis*-regulatory divergence to differences in expression between copies of duplicate genes in each pair. The 2-kb (from -1.5 kb to +0.5 kb) genomic regions surrounding TSSs were used as putative *cis*-regulatory sequences and their divergence was estimated with REALIGNER [37]. For genes with multiple TSSs, a TSS supported by the highest number of CAGE/PET tags was selected. This analysis was limited to group B duplicate genes with completely similar structures (a total of 158 duplicate gene pairs). We found a significant positive correlation (R = 0.242, *P *< 0.01) between the proportion of aligned sequences in the *cis*-regulatory region (*P*_*cis*_) and *R*_*expression*_. This implies that the divergence of *cis*-regulatory regions leads to expression divergence in duplicate genes. After duplicate genes created by retrotransposition (a total of 23 gene pairs) were excluded, the correlation coefficient was even higher (R = 0.252, *P *< 0.01). Through comparison between *K*_*S *_(which may serve as a neutral proxy, although see [38]) on the one hand and the proportion (corrected for multiple hits using HKY85 model) of aligned sequences in the *cis*-regulatory region on the other hand in each non-retrotransposed duplicate gene pair, we estimated whether the *cis*-regulatory regions evolved neutrally. We found that for 107 out of 135 duplicate gene pairs compared, *K*_*S *_was significantly higher (*P *< 0.001, Wilcoxon signed-rank test) than the proportion of aligned sequences in the *cis*-regulatory region, suggesting that purifying selection acts at *cis*-regulatory regions.

To investigate whether copy-specific TEs influence divergence in duplicate gene expression, we identified such TEs (TEs that integrated in the *cis*-regulatory region of only one duplicate gene copy of a pair after duplication) in the same 2-kb regions surrounding TSSs of the above 158 duplicate genes pairs (excluding 23 retrotransposed duplicate pairs; see Materials and methods). However, no significant correlation was found between the proportion of copy-specific TEs and either *P*_*cis *_for duplicate genes or *R*_*expression *_(data not shown). This suggests that the effect of copy-specific TEs on divergence in duplicate gene expression may be at best minor, although this issue requires additional studies.

### Interplay of multiple predictors in explaining divergence of paralogous gene expression

Because several factors studied above might be interrelated, we conducted multiple regression analysis to estimate the relative contribution of each factor to explaining the total variability in *R*_*expression*_. A total of four continuous predictors (*K*_*A*_, *K*_*S*_, the *K*_*A*_/*K*_*S *_ratio, and divergence of *cis*-regulatory sequences (labeled 'Cis') and three categorical predictors (shared versus not shared TSSs (labeled 'TSS'); completely versus incompletely similar gene structure (labeled 'Structure'); and tandem versus non-tandem gene organization (labeled 'Tandem')) as well as all possible pairwise interaction terms were used to build a regression model. After pruning nonsignificant terms, the final multiple regression model explained approximately 10% of the variation in *R*_*expression *_and consisted of eight predictors (Table 2). Five of these predictors remained significant after applying Bonferroni correction for multiple tests (Table 2). These predictors included: Tandem, TSS, and interaction terms between Structure and Tandem, between TSS and Tandem, and between *K*_*A*_/*K*_*S *_ratio and Cis (Table 2). Our computation of the relative contribution of the variability explained (RCVE) for significant predictors (see Materials and methods for details) indicated that each of them makes a sizeable input into the model.

**Table 2:** Multiple regression models for expression divergence in duplicate genes

Predictors	*P*-value	RCVE*
Cis^†^	4.2 × 10^-2 ^(NS^‡^)	0.075
TSS^§^	9.9 × 10^-5^	0.277
Tandem^¶^	2.7 × 10^-6^	0.405
*K*_*A *_× Cis	1.1 × 10^-2 ^(NS)	0.118
*K*_*S *_× Cis	2.7 × 10^-2 ^(NS)	0.088
Structure^¥ ^× Tandem	1.7 × 10^-3^	0.180
TSS × Tandem	1.1 × 10^-5^	0.354
ω^# ^× Cis	3.1 × 10^-3^	0.159
R^2^		0.093

*RCVE: relative contribution to the variability explained (see Materials and methods for more details). ^†^Cis: divergence of *cis*-regulatory sequences in 2 kb surrounding TSS (see Materials and methods for more details). ^‡^NS: not significant after Bonferroni correction for multiple tests. ^§^TSS: shared versus not shared TSSs. ^¶^Tandem: tandem versus nontandem organization of duplicate genes. ^¥^Structure: structural heterogeneity in coding sequences. ^#^ω:*K*_*A*_/*K*_*S *_ratio.

## Discussion

Although it has been shown that duplicate genes diverge rapidly in their expression [10,39-41], little is known about which factors influence their expression divergence at the genomic level [42]. In this study, we investigated three such factors: structural heterogeneity of coding sequences, turnover of TSSs, and divergence of *cis*-regulatory regions (including insertions of copy-specific TEs).

Our results indicate that structural differences in coding sequences are common among human duplicate genes. We observed a high proportion of duplicate genes with structural differences even among young duplicates (*K*_*S *_<0.1), which is consistent with the findings for *C. elegans *duplicate genes [15]. Thus, genes might already be structurally different at the point of duplication. In general, duplication by unequal crossing over might not contain the entire coding sequence of a parental gene, and indeed, for the majority of individual young duplicate gene pairs with incompletely similar structures in our data set (for approximately 90% of duplicate pairs of group A), both copies reside on the same chromosome. Over time, duplicate genes accumulate mutations leading to amino acid changes, premature stop codons, and atypical splicing [4,14,43]. These mutations might lead to decreasing numbers of duplicate genes retaining their ancestral structure and lead to more rapid divergence in expression and function.

Alteration of TSSs between duplicate gene copies is likely to have a direct impact on expression divergence. Using sequence similarity analysis, we examined whether duplicate genes share their TSSs. A large number of duplicate genes with distinct TSSs between the two copies were observed and these duplicate gene copies usually had different expression patterns. Although we did not directly estimate the fitness effects of turnover of TSSs on retention of duplicate genes, alteration of TSSs provides a means for the realization of several models of gene duplication evolution (for example, subfunctionalization and neofunctionalization [44,45]).

Additionally, we observed that *cis*-regulatory regions of duplicate genes diverge with time since duplication. This is consistent with several previous reports [46-48]. We investigated a potential impact of the density of copy-specific TEs on the divergence of duplicate gene expression and, surprisingly, found no major effect. This result corroborates recent findings regarding orthologous mammalian promoters; in human core promoters, the density of most observed repeat classes was significantly below the genomic average, suggesting that insertion of TEs in *cis*-regulatory regions is prevented by purifying selection [36].

Using multiple regression analysis, we observed that shared versus not shared TSS ('TSS'), completely versus incompletely similar structure ('Structure'), divergence of *cis*-regulatory sequences ('Cis'), the *K*_*A*_/*K*_*S *_ratio, and tandem versus non-tandem duplicate gene organization played an important role in determining divergence in duplicate gene expression. It is worth noting that all three novel predictors introduced in this manuscript (TSS, Structure, and Cis) significantly influence divergence in duplicate gene expression alone and/or through interaction with other predictors. Interestingly, *K*_*S*_, a proxy of evolutionary time, was not a significant predictor in our model. However, as noted above, evolutionary time influences alterations in other predictors and, therefore, the influence of *K*_*S *_on *R*_*expression *_might be observed through significance of predictors dependent on *K*_*S*_. While interaction terms are not straightforward to interpret, the finding that several of them significantly contributed to the model suggests that considering multiple correlated factors might be essential for understanding patterns of duplicate gene expression divergence.

In this study, expression pattern was used as an indicator of evolution of biological functions after gene duplication. Several studies have suggested that gene expression density and breadth (for example, in housekeeping versus tissue-specific genes) has significantly influenced the evolution of proteins [49-52]. In addition to gene expression, which is likely a strong predictor [53,54], several additional factors have been implicated in protein evolution. Such factors include gene dispensability [55,56], protein stability and interaction network [57,58] as well as codon usage [54,59]. Although these variables individually explain only a small fraction of variation in the rate of protein evolution, studying them might provide important insights into divergence between duplicate genes.

Most gene evolution models have assumed that two duplicate gene copies are expressed equally immediately after duplication. However, similarly to coding sequences, promoter regions might also be incompletely duplicated between copies; this possibility needs to be evaluated in future studies. Frequently, because of the complex evolutionary dynamics of promoter sequences [47,60,61], it is difficult to distinguish incomplete promoter duplication from rapid promoter evolution after duplication.

Reconstruction of ancestral gene expression state can be performed using a parsimony-based procedure in multi-gene families [62], instead of using the pairwise analysis employed here. However, rigorous filtering for potential cross-hybridization of transcripts of genes from the same multi-gene family in our study makes such ancestral reconstruction difficult. Thus, additional studies using different types of expression data may allow us to decompose the expression divergence of genes in multi-gene families and thus provide us with additional methodological insights for understanding gene expression divergence.

In the present study, as expected, we observed a significant negative correlation between the synonymous rate and Pearson correlation coefficient of expression values between duplicate gene copies; however, the resulting correlation was weaker than in our previous study [10]. There might be several potential reasons explaining this difference (for example, different *K*_*S *_thresholds used in the two studies and a greater number of tissues used in the present study). However, the major advance of the present study compared with the previous one [10] is a more rigorous filtering for potential cross-hybridization of transcripts of two duplicate gene copies to the same probe, and thus we consider the present results more robust.

## Conclusion

The present study represents the first report of the effects of structural differences in coding region and of unique TSSs on the divergence of duplicate gene expression. Our observations of frequent turnover of TSSs between duplicate genes and a high proportion of young duplicate genes with incompletely similar structures contradict the assumptions of classic gene duplication models, according to which duplicate genes are considered to be equal both structurally and functionally at the point of duplication [4,13,14]. Although potential incomplete duplication of promoters will be the subject of future studies, our investigation of factors contributing to expression divergence of duplicate genes provides important information for understanding human transcriptome heterogeneity, complexity, and evolution.

## Materials and methods

### Identification of duplicate gene pairs

To cluster genes into families, we downloaded 48,218 protein sequences of consensus coding sequences, known and novel genes from Ensembl (release 38 of NCBI build 36) and independently used the FASTA [63] and TRIBE-MCL [64] methods to define duplicate gene families. Briefly, for the FASTA method, each protein sequence was used as a query to search against all other protein sequences using FASTA [65] with E < 10. Two protein sequences formed a link if: the aligned region was >80% of the longer protein; and the identity between two proteins was ≥ 30% for alignments longer than 150 amino acids or ≥ (0.01n + 4.8L^-0.32 [1+exp(-L/1000)]^) otherwise, where *L *is the alignable length between two proteins and n = 6. The formula above was derived from empirical data, which suggested that a higher sequence identity was required for shorter proteins [66]. These gene pairs were grouped into gene families according to the single linkage clustering algorithm. For gene families derived by TRIBE-MCL, we downloaded the gene annotations through BioMart in the Ensembl database, and considered gene families with at least two members.

To identify independent pairs of duplicate genes within each gene family, we sorted gene pairs in ascending order of *K*_*S *_and selected the pair with the lowest *K*_*S*_. After excluding genes that had been picked, we chose the next gene pair with the lowest *K*_*S*_. These steps were repeated for each gene family. All genes encoding proteins were realigned using CLUSTALW [67], and the yn00 module [68] of PAML [69] was used to calculate *K*_*S*_. We counted duplicate gene pairs in intervals of size *K*_*S *_= 0.01 to derive the instantaneous rate of *K*_*S*_ according to [35].

Duplicate gene copies in which one of the pair has one exon and the duplicate copy has multiple exons were called retrotransposed duplicate gene copies. In addition, duplicate gene pairs were classified as tandem duplicates if there were no genes separating them.

### Expression data analysis

Expression data for 61 non-redundant and nonpathogenic human tissues in U133A and GNF1H Affymetrix arrays were obtained from [28]. To validate mapping between probe sets and genes, we aligned the transcripts of consensus coding sequences, known genes, and novel genes downloaded from Ensembl (release 38 of NCBI build 36) with the exemplar and consensus sequences for each array using BLAST [70] with E < 10^-20^. According to the criteria described in [71,72], the acceptable alignments were selected if: the identity was 100% and the length was greater than 49 bp; or the identity was higher than 94% and the length was at least either 99 bp or 90% of the length of the query. We considered three scenarios for mapping relationships: a single probe set hitting one gene (9,508 probe sets); multiple probe sets hitting one gene (13,186 probe sets and 4,997 genes); and a single probe set hitting multiple genes (4,493 probe sets and 6,764 genes). All genes following the first two scenarios were utilized in the present study. For each gene following the second scenario, the probe set with the highest expression value (defined by average difference) was selected. All genes following the third scenario were removed from the analysis due to potential cross-hybridization. Following [28], genes with average difference >200 in a particular tissue were considered to be expressed in this tissue.

### Identification of putative TSSs

The putative TSSs were identified using the method described in the ENCODE pilot project [73]. Briefly, we utilized tag clusters from two sets of 5'-end-tag-capture technologies: CAGE [20] and PETs [31]. If two tag clusters were located on the same strand and within 60 bp (which was derived from analyzing the distribution of distances between tag clusters in [73]) of each other, they were considered as one tag cluster. To map tag clusters to genes, the following two criteria were considered. First, the strand of a tag cluster was required to be identical to the strand of a gene. Second, a tag cluster was required to be located in the 5' upstream region from the most upstream start codon of a gene. Because we constructed artificial coding regions of genes by including all their exons, our analysis is not affected by alternative start codons. To confirm the reliability of the tag data, RefSeq [74], H-Invitational [75] and human ESTs [76] RNA data from the UCSC Genome Browser [77] were utilized. We excluded tag clusters with a single tag as well as those whose coordinates did not overlap with the genomic coordinates of the 5' end of cDNAs or ESTs. To define a representative tag site (to be used as a putative TSS) for each tag cluster, we selected the tag site that was supported by the highest number of 5' start sites. Otherwise, if several sites in a tag cluster had the same number of 5' start sites, the central coordinate of this tag cluster was defined as the representative tag site.

### Analysis of turnover of TSSs between human-mouse orthologous gene pairs

To evaluate conservation of TSSs between human-mouse orthologous genes, we obtained two distinct classes of orthologous genes from [23]. Briefly, 'conserved promoter regions' means that upstream sequences of TSSs between human and mouse orthologous genes were aligned; otherwise, 'non-conserved promoter regions' means there were no significant alignments. We excluded orthologous genes that were classified into both classes because alternatively spliced variants of each gene had different conservation patterns of promoter regions. As a result, 1,610 orthologous gene pairs that were classified into just one class in a mutually exclusive manner were retained. We downloaded human and mouse protein sequences from Ensembl (release 38 of NCBI build 36). All genes were aligned using CLUSTALW [67], and the yn00 module [68] of PAML [69] was used to calculate *K*_*S *_between orthologous genes.

### Classification of the type of gene duplication into structural categories

Structural categorization of duplicate genes was performed using reconstructed full-length coding sequences. We downloaded annotated human genome data from Ensembl (release 38 of NCBI build 36). Alternatively spliced variants lacking start or stop codons or lacking canonical exon boundaries (5'-GT...AG-3', 5'-GC...AG-3', or 5'-AT...AC-3') were excluded. For each gene with several alternatively spliced variants, all exons were aligned against each other, and, if some exons overlapped, they were merged in a single exon. Next, exons were sorted by their genomic coordinates and were reassembled to form reconstructed full-length coding sequences.

The reconstructed full-length coding sequences were aligned using AVID [78] with default parameters. Each pair of duplicate genes was classified into one of the four structural categories: completely similar, 5' similar, 3' similar, and neither 5' nor 3' similar. If the proportion of aligned sequences was greater than 0.9, duplicate gene pairs were categorized as completely similar. The other duplicate gene pairs were exclusively classified in just one category of 5' similar, 3' similar, or neither 5' nor 3' similar. If alignments between the two copies started at the start codons of both copies, then such duplicates were classified as 5' similar. Alternatively, if the alignments ended at the stop codons of both copies, we classified the duplicate genes into 3' similar. Finally, the remaining duplicate gene pairs were labeled as neither 5' nor 3' similar.

### *Cis*-regulatory regions analysis

To detect homologous sequences in *cis*-regulatory regions, we used a modified version of REALIGNER [37]. Using BL2SEQ (part of the Blast suite [70]) with mismatch penalty equal to -2 and word size equal to 7, we constructed alignments of 2-kb (-1.5 kb to +0.5 kb) genomic regions surrounding putative TSSs between copies in each duplicate gene pair. We selected alignments satisfying three criteria: hit length >7 bp; identity >70%; and identical hit strand. If two local alignments overlapped, an alignment with the higher bit score was retained. If the bit scores of the two overlapping alignments were identical, a longer alignment or the one closest to TSS was retained. If the two local alignments were not syntenic (the order of blocks in each alignment was inconsistent), an alignment with the lower bit score was removed. Finally, all local alignments ordered by their genomic coordinates were used as a conserved *cis*-regulatory region for a duplicate gene pair.

TEs within *cis*-regulatory regions were classified into two sets: with the insertion occurring in the ancestral sequence before duplication of a genomic region; with the insertion in only one duplicate copy after the duplication event. We used the Repeatmasker [79] tables at the UCSC Genome Browser [77] to map the coordinates of TEs into *cis*-regulatory regions.

### Multiple regression analysis

Linear multiple regression analysis was performed in the R statistical package. The original model included all seven predictors and their interaction terms, but was pruned to include only significant predictors (and significant interaction terms). RCVE [80,81] was utilized to assess the contribution of each predictor to explaining the total variability:

$$RCVE=\frac{R_{full}^{2}-R_{reduced}^{2}}{R_{full}^{2}}$$

where $R_{full}^{2}$ and $R_{reduced}^{2}$ are the *R*^2 ^for the full model and the model except for the predictor of interest, respectively. In addition, variance inflation factors [82] were calculated for each predictor to diagnose multicollinearity. All predictors and their interaction terms included in the final model had variance inflation factors below 2 (data not shown), suggesting that multicollinearity was not adversely affecting the model.

## Abbreviations

CAGE: cap analysis of gene expression; *K*_*A*_: nonsynonymous divergence; *K*_*S*_: synonymous rate; PET: paired-end ditag; RCVE: relative contribution to variability explained; TE: transposable element; TSS: transcription start site.

## Authors' contributions

CP and KDM designed the experiments and wrote the manuscript. CP performed data analyses.

## Additional data files

The following additional data are available with the online version of this paper. Additional data file 1 is a table listing the classification of duplicate gene pairs based on the absence or presence of shared TSSs and different duplication mechanisms. Additional data file 2 is a Venn diagram depicting the number of duplicate gene pairs that were identified by the FASTA and TRIBE-MCL methods. Additional data file 3 shows average sequence identity between TSS regions of duplicate genes. Additional data file 4 shows number of duplicate gene pairs with shared TSSs (A) and without shared TSSs (B) plotted against the instantaneous rate of *K*_*S*_. Additional data file 5 shows proportions of group A duplicate gene pairs with shared TSSs depending on different identity thresholds. Additional data file 6 shows number of duplicate gene pairs in different structure categories plotted against the instantaneous rate of *K*_*S*_.

## Supplementary Material

Additional data file 1 Classification of duplicate gene pairs based on the absence or presence of shared TSSs and different duplication mechanisms. Click here for file

Additional data file 2 Number of duplicate gene pairs that were identified by the FASTA and TRIBE-MCL methods. Click here for file

Additional data file 3 The identities were obtained by BL2SEQ [70] with default parameters. Black bars represent 110 bp (-20 bp to +90 bp) surrounding each TSS. Click here for file

Additional data file 4 Number of duplicate gene pairs (A) with shared TSSs and (B) without shared TSSs plotted against the instantaneous rate of *K*_*S*_. Click here for file

Additional data file 5 Proportions of group A duplicate gene pairs with shared TSSs depending on different identity thresholds. Click here for file

Additional data file 6 Number of duplicate gene pairs in different structure categories plotted against the instantaneous rate of *K*_*S*_. Click here for file
